# Relationship between liver lipid and liver dry matter in slaughtered ruminants

**Published:** 2012

**Authors:** Hossein Tajik, Aligholi Ramin, Shahram Nozad, Babak Jelodari, Ghazaleh Ashtab, Zohreh Eftekhari, Sina Ramin

**Affiliations:** 1 *Department of Food Hygiene and Quality Control, Faculty of Veterinary Medicine, Urmia University****, ****Urmia Iran; *; 2 *Department of Clinical Sciences, Faculty of Veterinary Medicine, Urmia University****, ****Urmia Iran; *; 3 * Department of Clinical Sciences, Faculty of Veterinary Medicine, Tehran University****, ****Tehran, Iran; *; 4 *Tabriz University of Medical Sciences, Tabriz, Iran.*

**Keywords:** Lipidosis, Ruminants, Sex, Age, liver

## Abstract

Lipids in liver wet and dry matter, liver moist and dry matter and their relationships were investigated based on species, sex and age in cows, buffaloes, sheep and goats. Mean percentage of lipids in liver wet and dry matter and liver dry matter in cows were 3.60%, 1.10%, 29.70%, and for buffaloes were 5.30%, 1.55%, 29.20%, sheep 3.00%, 0.83%, 27.90%, and goats 2.910%, 1.55% and 28.40%, respectively. The highest and lowest percentage of lipids in liver wet and dry matter was observed in buffaloes and sheep, and for the liver dry matter was recorded in cows and sheep, respectively. Analyses showed significant differences in liver parameters among ruminants (*p* < 0.01). Gender, except for goats, did not affect the animals' liver parameters. In overall 15.00% of buffaloes and 3.50% of cows showed over 10.00% lipids in liver, while none of small ruminants appeared to have over 6.00% lipids in liver. There was no correlation between liver lipid and liver dry matter. In conclusion mean percentage of lipid in liver dry matter in small ruminants was less than large ruminants. Liver dry matter was high in cows and low in sheep. Mean differences in liver parameters was significant, while the age and sex of the animals were not. Liver lipidosis in buffaloes seems greater than in cows, and in small ruminants it was negligible. No correlation was expected between liver parameters. Finally, on the basis of liver dry matter, the liver in ruminants ranked from cows to buffaloes, goats and sheep.

## Introduction

Liver is an appendix of the gastrointestinal tract and its activity in the body is categorized as the production of energy, proteins, vitamins and toxic detoxification mechanism. Enzymes secretion, formation and excretion of bile salts and ingestion of lipids in intestine are also considered as other functions of this organ.^1^ However, the major function of liver is to produce energy from carbohydrates and fat reservoirs. Body lipids, as the main sources of energy, participate in liver structure and by processing glyconeogenesis, result in liver glycogen production and prevention of acetonemia.^2^ The detrimental effect of lipid accumulation in liver is very serious and lipidosis or fatty liver can disrupt the vital function of liver.^3^

The normal range for lipids in liver dry matter is less than 10.00%.^1^ The bibliography on changing the lipid in liver between different ages and sex is limited to feedlot and productive herds and the occurrence of fat liver syndrome in adult animals is more common than in young animals, but in some situations liver lipidosis occurs in young animals, sporadically.^4^^,^^5^ In pregnant animals, because of the negative energy balance after parturition, the fat transfers to the liver up to 20.00% of dry matter,^6^^, ^^7^ and then after milking it declines to 5.00% within 26 weeks after parturition.^1^

One of the effects of fat infiltration in liver is obesity syndrome, mainly in heifers and bulls. According to some reports, sex and age can have an effect on lipids in liver of ruminants and there are many investigations reaching fatty liver in feedlot and productive ruminants,^8^ however, little information exists in non-productive ruminants. Thus, this study was aimed at evaluating: 1) the percentages of lipids in liver wet and dry matter based on sex and age in cattle, buffaloes, sheep and goats 2) to determine the effect of lipids in liver dry matter 3) correlations between liver parameters on the basis on the ages and sex of animals 4) to determine the role of lipid in liver dry matter, as lipidosis can have an effect on frangibility of liver. 

## Materials and Methods

Three hundred and eighty eight slaughtered ruminants including 114 cattle (66 cows, 48 bulls), 100 buffaloes (44 buffalo cows, 56 buffalo bulls), 147 sheep (15 ewes, 132 rams) and 27 goats (3 does, 24 bucks) were selected. Animals were of different ages and the number of females and males were 128 and 256, respectively. Samples were prepared in overall 37 visits from Urmia abattoir. The age of the animals was determined by the presence of temporary and permanent incisor teeth. The breeds of selected ruminants were hybrid and Holstein for cows, and native breeds for buffaloes, sheep and goats.

The animals were examined before slaughter. At least 10 grams from the ventral part of the liver was prepared after they were slaughtered. The samples were transferred to food quality control laboratory, Faculty of Veterinary Medicine, Urmia University, Urmia, Iran in a special container. At first, 3 grams of liver was dried in an oven at 105 °C for 24 hours. The liver dry matter was calculated as shown below:

$\mathrm{Dry matter}=\frac{W^{1}˗W^{2}}{W^{1}}\times \mathrm{100}$

*W* ^1^ *=Initial weight, W* ^2^ *= Final weight.*

After calculating the liver dry matter, the percentage of liver humidity was also evaluated. The amount of lipid in liver dry matter was measured by Socksile test using the extraction of fat by N-Hexane solution and solvent extracted with evaporation and, after drying, the amount of extracted fat was estimate. For determination of lipids in liver wet matter, the liver was boiled in 10% diluted hydrochloric acid and then the substances were passed through filter (fiber) paper and dried. The remained substances on smooth paper were extracted with N-Hexane solution (Ziest Chimi Diagnostics, Tehran, Iran). 

Data were analyzed by SPSS software (version 18 for windows, SPSS Inc., Chicago, IL, USA) and mean ± SEM were determined for liver parameters based on age, sex and animal species. One way ANOVA and student *t*- test were carried out to find the differences in the parameters under study. Pearson’s correlation test was used to evaluate the relationships between lipids in liver dry matter and liver dry matter. A *p*-value less than 0.05 was considered to be significant.

## Results

**Fig. 1:**
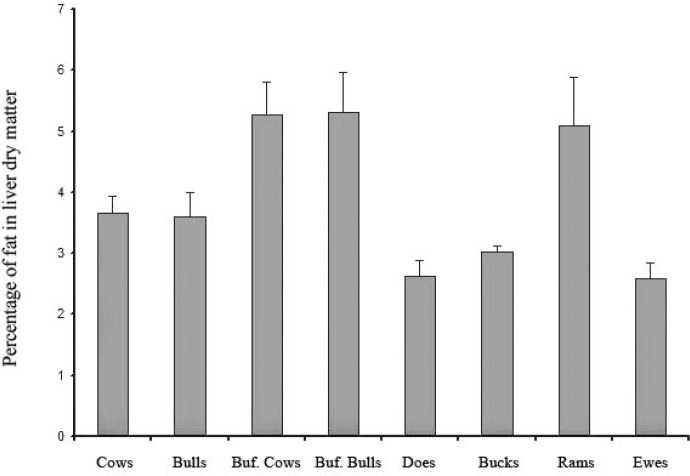
Comparison of mean ± SE percentage of fat in liver dry matter among the ruminants.

**Fig. 2:**
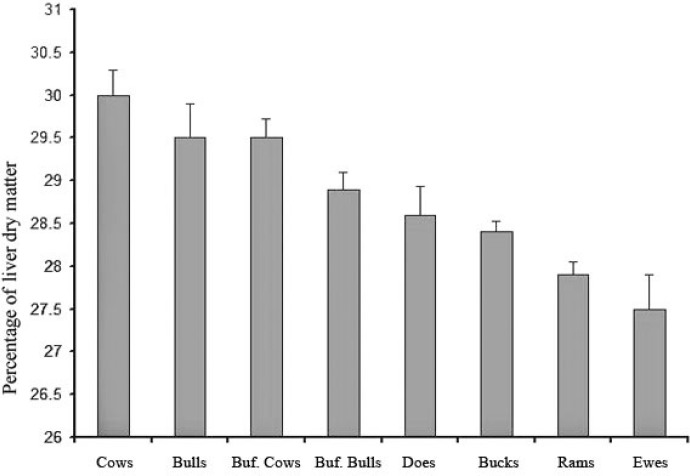
Comparison of mean ± SE of liver dry matter among the ruminants

According to Table 1, the highest and lowest mean percentage of lipid in liver dry matter was observed in buffaloes (5.29%) and bucks (2.59%), respectively (Fig. 1). The maximum and minimum mean liver dry matter in cows was (30.00%) and in ewes (27.50%), respectively (Fig. 2). Statistical analyses showed significant differences in liver parameters among ruminants (*p* < 0.01). The variations in liver lipids among ruminants were different and varied from 0.40% to 20.50% in buffaloes, and for liver dry matter were from 22.30% to 38.20%. There were no sex and age differences in liver parameters except for goats, in which the value for does was higher than ducks. After pooling the data, the values for liver lipids in buffaloes were greater than others. In overall 15.00% of buffaloes and 3.50% of cows were revealed to have over 10.00% lipid in liver, while small ruminants had less than 6.00% lipid in their liver. The results of correlations between liver parameters among ruminants showed that in spite of very reliable and strong relationships between lipids in wet and dry matter of liver, no significant correlation existed between lipids in liver and liver dry matter.

**Table 1:** Comparison of mean ± SE and the range of liver parameters in cows, buffaloes, sheep and goats (n=388).

**Liver parameters**	**Percentage of fat in liver dry matter**		**Percentage of fat in liver wet matter**		**Liver Dry matter**	
**Animals**	Mean ± SE	Range	Mean ± SE	Range	Mean ± SE	Range
**Cows**	3.65 ± 0.29^a^	0.40 - 11.40	1.10 ± 0.90^a^	0.10 - 3.50	30.00 ± 0.30^a^	23.00 - 38.20
**Bulls**	3.60 ± 0.40^a^	0.70 - 12.40	1.00 ± 0.10^a^	0.20 - 3.60	29.50 ± 0.40^a^	22.30 - 35.20
**Cattle**	3.60 ± 0.22^a^	0.40 - 12.40	1.10 ± 0.60^a^	0.10 - 3.60	29.70 ± 0.23^a^	22.30 - 38.20
**Buffaloes bulls**	5.27 ± 0.54^b^	0.70 - 13.90	1.54 ± 0.20^b^	0.20 - 3.90	29.50 ± 0.22^a^	26.70 - 34.10
**Buffaloes cows**	5.30 ± 0.66^b^	0.40 - 20.50	1.55 ± 0.20^b^	0.10 - 6.2	28.90 ± 0.20^a^	26.00 - 33.00
**Buffaloes**	5.30 ± 0.44^b^	0.40 - 20.50	1.60 ± 0.13^c^	0.10 - 6.20	29.20 ± 0.15^a^	26.00 - 34.10
**Ewes**	2.63 ± 0.26^c^	0.98 - 4.20	0.72 ± 0.70^a^	0.28 - 1.10	27.50 ± 0.33^b^	25.90 - 29.50
**Rams**	3.03 ± 0.09^a^	0.92 - 5.14	0.84 ± 0.30^a^	0.26 - 1.55	27.90 ± 0.13^b^	24.30 - 31.90
**Sheep**	3.00 ± 0.90^a^	0.92 - 5.14	0.83 ± 0.20^b^	0.26 - 1.55	27.90 ± 0.12^b^	24.30 - 31.90
**Does **	5.10 ± 0.79^b^	3.90 - 6.60	1.48 ± 0.30^c^	1.00 - 2.06	28.60 ± 0.15^ac^	25.90 - 31.10
**Bucks **	2.59 ± 0.26^c^	0.80 - 5.30	0.80 ± 0.73^c^	0.22 - 1.49	28.40 ± 0.40^ac^	26.50 - 32.40
**Goats**	2.91 ± 0.30^c^	0.80 - 6.60	0.90 ± 0.83^a^	0.22 - 2.06	28.40 ± 0.40^ac^	25.90 - 32.40

## Discussion

The highest percentage of lipids in liver dry matter in high producing cows and ketosis was reported to be up to 70.00%. In this study, small ruminants (2.29%) showed the lowest percentage of liver lipid compared to cattle (3.60%) and buffaloes (5.29%). The normal value for lipids is less than 10.00% of liver dry matter and can rise up to 70.00% in different diseases in different species.^1^ Reports show 53.10% in cattle,^9^^,^^10^ 5.60% in Indian buffaloes,^2^ and 2.20 to 3.30% in sheep and goats.^11^^,^^12^ This information shows that liver lipids vary between different species of ruminants irrespective of sex and age. Lawrie,^13^ however, reported the impact of age and sex on cows as observed similarly for goats in this study. In an individual survey of liver lipid of animals, 3.50% of cows and 15.00% of buffaloes had over 10.00% lipid in their liver, while none of the small ruminants showed over 6.00% lipid in liver, meaning the incidence of liver lipidosis in large ruminants is more common than in small ruminants.^6^

Liver dry matter in sheep and goats (27.90%) was less than in buffaloes (29.20%) and cows (29.75%) as reported by Park *et al.* up to 24.10% and Mustafa up to 27.50%.^11^^,^^12^ By increasing the proteins, fats and carbohydrates in liver, humidity may decrease at the expense of dry matter. While, by increasing the fats only, the dry matter will decline. In this study there were no significant differences in liver dry matter in terms of age and sex. Determination of the liver dry matter can lead to understand the carbohydrates and lipids storage of liver, and this could be a reflection of nutritional management among animals. Dehydration and water loss of the body in different species have had an effect on the lipid reserves of the body and resulted in low dry matter.^9^

The ratio of liver humidity to dry matter in sheep (2.90), goats (2.50), cattle (2.38) and buffaloes (2.42) varied significantly (*p* < 0.05) among ruminants. The reports on liver humidity for sheep,^12^ goats,^11^ cattle,^9^ and buffaloes^2^ show higher values than this study, which means a lower content of liver lipids in Urmia ruminants compared to other countries. This ratio in the cows' ketosis rises up to 3. According to the literature, the quality of ruminants' liver depends indirectly on the low level of this ratio, which means that the nutritional quality of liver in ruminants is different among cattle, buffaloes, goats and sheep. Starvation and lack of suitable food will decrease the rate of humidity in liver.^14^ The storage of fats, osmolarity and oxidative metabolism and phospholipids could affect the hydration process of hepatocytes, resulting in low liver dry matter.^9^^,^^15^^-^^17^

According to the literature, the clinical incidence of metabolic fat cow syndrome is more common in cows than other ruminants,^18^^-^^21^ while nutritional liver lipidosis due to vitamin E and selenium, cobalt and iron deficiencies is more common in small ruminants than in cows.^22^^-^^24^ In other words, buffaloes, sheep and goats as non-high producing animals are more susceptible to nutritional disorders of liver lipidosis than the metabolic form of disease. In this study, the feedlot males were all young without visible disorders in their liver, thus these values for liver lipid would be considered the same as for healthy animals. Although gender did not affect liver lipids in ruminants and in spite of differences between does and bucks, as they were less than 5.00% lipid in liver, these differences were therefore considered as a normal condition and not pathologic pattern.

The presence of strong significant correlations between percentages of lipids in liver dry matter/wet dry matter and liver humidity/dry matter is reasonably expected, but the absence of a significant relationship between liver lipid and humidity or with dry matter could explain why lipid has no effect on liver dry matter in ruminants. This means that these two parameters are independent in health status,^9^ though in one older report a significant negative correlation between lipid and liver dry matter has been mentioned.^14^ It is concluded that liver lipid and dry matter in small ruminants was less than in cows and buffaloes. Sex and age of animals had no influence on liver lipid and no correlation was found between liver lipid and dry matter. The occurrence of liver lipidosis in buffaloes and cows is more common than in sheep and goats. Finally, based on the values of liver dry matter, the quality of liver in ruminants can be sorted down from cows to buffaloes, goats and sheep. 
